# Visual Outcomes of Early Intravitreal Antibiotics and Factors Affecting Presumed Acute Endophthalmitis Post Cataract Surgery in Tertiary Eye Hospital: An Observational Study

**DOI:** 10.31729/jnma.9179

**Published:** 2025-08-31

**Authors:** Lily Rajbanshi, Ananya Singh, Kabma Shreflha, Reema Niraula, Pragya Luite, Sristi Thakur

**Affiliations:** 1 Department of Vitreo-Retina, Biratnagar Eye Hospital, Biratnagar, Morang, Nepal

**Keywords:** *acute endophthalmitis*, *cataract surgery*, *intravitreal antibiotics*, *visual outcomes*

## Abstract

**Introduction:**

Acute pofl-cataract infectious endophthalmitis is a rare yet serious complicationthat can lead to significant intraocular inflammation and potential loss of vision following cataract surgery. Early diagnosis and prompt treatment are essential to prevent irreversible damage to photoreceptors. While the Early Vitrectomy Study (EVS) provided initial treatment guidelines, clinical approaches have since evolved.

**Methods:**

In this observational, retrospective single-center study at Biratnagar Eye Hospital, we analyzed data from patients diagnosed with presumed endophthalmitis within six weeks of cataract surgery in 2023. All patients received intravitreal antibiotics (vancomycin and ceftazidime) and underwent tap biopsy for microbiological analysis. Data included demographics, clinical features, treatment (intravitreal antibiotics ± vitrectomy), and visual acuity (LogMAR) at baseline and 60 days. The data were recorded in Excel and analyzed using Statical Package for the Social Sciences version 29.0 (IBM Corp., Armonk, NY, USA).

**Results:**

The study found that 44 (87.70%) were between 40-80 years old, with males 28 (57.14%). Symptoms appeared primarily within the firfl two weeks pofl-surgery in 31 (62%) of cases. Phacoemulsification 24 (48%) and Small Incision Cataract Surgery 26 (52%) were represented in the patient population. Out of total patients,44 (88%) of the patients received only intravitreal antibiotics. A notable improvement was observed in visual acuity with mean Log MAR values decreasing from 1.395± 0.632 on Day 1 (Snellen equivalent 6/120) to 0.441±0.553 on Day 60 (Snellen equivalent 6/15). No notable variations in visual acuity was observed in age, gender, surgical technique (phacoemulsification vs. SICS), or adjunctive dexamethasone.

**Conclusions:**

Findings suggefled that early therapeutic intervention with intravitreal antibiotics in acute pofloperative endophthalmitis may improve visual outcomes when evaluated at 60 days pofl-treatment.

## INTRODUCTION

Acute endophthalmitis is a serious, sight-threatening complication of cataract surgery. Presumed acute endophthalmitis is defined as a clinical diagnosis of acute inflammation caused by a suspected bacterial/fungal infection based on symptoms including sudden loss of vision, ocular pain, red eye, Anterior chamber (AC) cells and flare, hypopyon and vitreous cells clumping occurring within 6 weeks.^1-3^ It affects 0.02% and 0.1% of cataract surgeries, with a substantial proportion of cases resulting in poor visual outcomes.^4,5^ The infection can be caused by various pathogens, including Grampositive and Gram-negative bacteria, as well as fungi.^6^ Early treatment with intravitreal antibiotics is crucial for improving visual prognosis, especially when administered within 24-48 hours of symptom onset.^7,8^

This study examines the effect of early intravitreal antibiotic administration in managing acute endophthalmitis subsequent to cataract surgery. Through systematic analysis of patient outcomes, the study seeks to identify the predictive factors for visual recovery and improve treatment strategies for better visual prognosis.

## METHODOLOGY

This retrospective observational study was conducted at a single tertiary care center (Department of Retina OPD, Biratnagar Eye Hospital, Rani) including patients who had undergone cataract surgery between January 1 and December 31, 2023 following ethical clearance from Institutional Review Committee, Biratnagar Eye Hospital (Reference no: 108/2024) The study population comprised consecutive cases of presumed postoperative endophthalmitis following cataract surgery and subsequently received combined medical and surgical management at the institution. We identified 50 cases of suspected post-cataract endophthalmitis from a total pool of 42297 cataract surgery patients during the study period.

The study included adult patients who had undergone cataract surgery at Biratnagar Eye Hospital between January and December 2023 and had subsequently presented to the retina outpatient department (OPD) with clinical features suggestive of endophthalmitis. These patients had received treatment with intravitreal antibiotics, either alone or in combination with dexamethasone, and had returned for follow-up visits up to 60 days post-treatment. Patients were excluded if they had developed endophthalmitis following ocular procedures other than cataract surgery (such as pars plana vitrectomy or trabeculectomy), had post-traumatic endophthalmitis, had presented with symptoms beyond six weeks after surgery, or if their initial cataract surgery had been performed at a facility other than Biratnagar Eye Hospital.

A pretested and self-adapted questionnaire from previous literature was used to collect relevant data for analysis. Data included demographic details, type of cataract surgery, visual acuity before surgery, surgical complications, time of presentation of patient to the hospital, sign and symptoms, type of intravitreal injection (vancomycin and ceftazidime), need of dexamethasone, follow up visual acuity and any other procedure needed.

Various clinical sign and symptoms of the patients such as pain, conjunctival congestion, hypopyon and anterior chamber reaction were noted. Pain intensity was recorded using an 11-point Numerical Rating Scale (NRS; 0=no pain, 10=worst pain imaginable).^9^ Conjunctival congestion was graded according to the standardized CCLRU scoring system (0=no conjunctival hyperemia to 3-4 = severe vascular congestion).^10^ Hypopyon was documented as presence/absence and measured in millimeters using slit-lamp micrometry; mobility and color characteristics were also noted. Anterior chamber inflammation was graded using the Standardization of Uveitis Nomenclature (SUN) criteria: AC cells (0: <1 cell, 0.5+: 1-5, 1+: 6-15, 2+: 1625, 3+: 26-50, 4+: >50) using a 1 mm x 1 mm slit beam.^11^ A change in inflammation was considered clinically meaningful if the grade changed by ≥2 steps (or 1 step when moving from 0.5+ or 3+ to 4+).

Vitritis, or inflammation of the vitreous humor, was graded using the Nussenblatt scale, which is incorporated into SUN guidelines for posterior segment inflammation.^12^

Fibrinous reaction refers to the presence of fibrin strands or coagulum in the anterior chamber, indicating intense intraocular inflammation. Unlike anterior chamber cells or flare, fibrin was not quantified in a graded manner in the original SUN criteria, but its presence was noted qualitatively due to its clinical significance. Severity was implied by descriptors such as mild fibrin strands, moderate fibrin web, severe fibrin clot, very severe or dense fibrin clot. The data were recorded in an Excel spreadsheet and analyzed using the Statistical Package for the Social Sciences version 29.0 (IBM Corp., Armonk, NY, USA).

Categorical variables were summarized as frequencies and percentages, while numerical data were expressed as means and standard deviations. Visual acuity measurements were converted from Snellen values to LogMAR for analytical purposes. Data distribution was assessed using histograms, Q-Q plots, and box plots, with skewness and kurtosis values verified to fall within the range of -2 to +2.

## RESULTS

**Figure 1:**
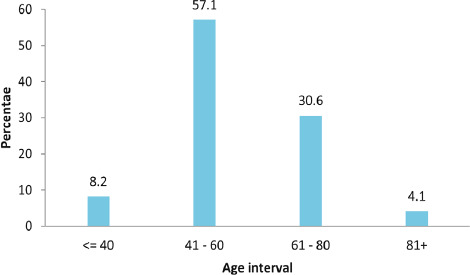
Timing of presentation after cataract surgery in patients with acute endophthalmitis (n=50).

Out of 50 patients included in the study, 29 (58%) were in the 41-60 years age group and 15 (30%) were in the 61-80 years age group (Figure 1).

The clinical presentation in the study cohort (N = 50) showed pain severity as mild in 31 (62%) and moderate in 14 (28%), with no severe cases. Conjunctival congestion was graded as mild in 16 (32%), moderate to severe in 28 (56%), and very severe in 1 (2%). Lid swelling was present in 5 (10.0%). Hypopyon was absent in 19 (38%), Grade 1 in 17 (34.0%), Grade 2 in 13 (26%), and Grade 3 in 1 (2%).

Anterior chamber reaction was moderate in 27 (54%), severe in 16 (32%), very severe in 4 (8%), and absent in 1 (2%). Corneal clarity was maintained in 42 (84.0%), while 8 (16%) had changes: Descemet’s membrane folds 2 (4.0%), corneal opacity 2 (4%), moderate edema 1 (2%), corneal scar 1 (2%), adherent leucoma 1 (2%), and hazy cornea 1 (2%). Vitreous status was poor in 17 (34%). Vitritis was graded as 1+ in 3 (6%), 2+ in 9 (18.0%), 3+ in 8 (16%), and 4+ in 13 (26%). Fibrinous reaction was present in 46 (92%): mild in 15 (30%), moderate in 18(36%), severe in 8(16%), and very severe in 5 (10%); absent in 4 (8%).

**Table 1:** Treatment Details and Visual Outcomes (n=50)

Variables	n(%)
Type of Surgery	
Phacoemulsification	24(48)
SICS	26(52)
Intravitreal Antibiotic Administration	
Only IV Antibiotic	44(88)
IV AB + IV Dexamethasone	6(12)
Pars Plana Vitrectomy	
Yes	16(32)
No	34(68)

SICS= Small Incision Cataract Surgery; IV AB = Intravitreal Antibiotic; IV Dexamethasone= Intravitreal Dexamethasone.

Out of 50 patients with acute endophthalmitis following cataract surgery, phacoemulsification was performed in 24 (48%) and small incision cataract surgery (SICS) in 26 (52%). Intravitreal antibiotics were administered in 44 (88%), while 6 (12%) received intravitreal antibiotics with dexamethasone. Pars plana vitrectomy was performed in 16 (32%) and was not performed in 34 (68%) (Table 1).

**Figure 2:**
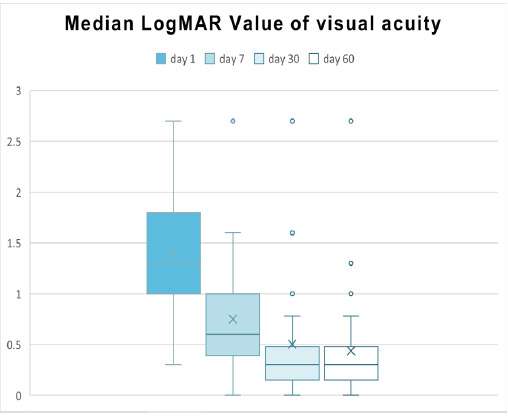
Box and Whisker Plot of LogMAR visual acuity over time (n=50).

The box and whisker plot (Figure 2) shows the distribution of visual acuity in LogMAR values at four time points: Day 1, Day 7, Day 30, and Day 60. The median values at each follow-up are presented.

**Table 2:** Changes in visual acuity after intravitreal antibiotic injection (n=50).

Examination day	N	Mean±SD
Day 1	50	1.395±0.63
Day 60	15	0.441±0.55

On Day 1, the mean visual acuity was 1.395 LogMar (SD = 0.632), while by Day 60, it improved to a mean of 0.441 LogMar (SD = 0.553) indicating a substantial improvement in visual outcomes over the 60-day period.

**Table 3:** Distribution of Visual Prognosis in Endophthalmitis Patients Post-Cataract Surgery by various demographic and clinical variables (n=50).

Independent factors	Log MAR ≤ 1 Good n (%)	Log MAR >1 Poor n (%)	Total
Age			
20-40	03(100)	00(0.0)	3
41-60	25(86.21)	4(13.79)	29
61-80	15(93.75)	1(6.25)	16
81-100	2(100)	00(0.0)	2
Gender			
Male	20(90.91)	2(9.09)	22
Female	25(89.29)	3(10.71)	28
Types of surgery			
Phacoemulsification	21(91.30)	2(8.70)	23
SICS	24(88.89)	3(11.11)	27
Days of presentation after surgery			
0-2 week	29(90.63)	3(9.37)	32
2-4 week	10(83.33)	2(16.67)	12
4-6 week	5(100)	00 (0.0)	5
Lid swelling			
Present	1(20)	4(80)	5
Absent	37(82.22)	8(17.78)	45
Only IV Antibiotic	41(89.13)	5(10.87)	46
IV antibiotic and IV Dexamethasone	3(75)	1(25)	4

The study included 50 cases of acute postoperative endophthalmitis following cataract surgery. The mean age of patients was 58.4±12.7 years, with 28 (56%) males. A total of 31 (62%) cases presented within 0-2 weeks after surgery. Anterior chamber reaction was moderate to severe in 43 (86%) cases, and hypopyon was observed in 31 (62%) cases (Grade 1-3). Vitreous involvementwas present in 33 (66%), with 13 (26%) cases showing 4+ vitritis. Corneal clarity was maintained in 42 (84%). Intravitreal antibiotics (vancomycin 1 mg/0.1 ml and ceftazidime 2.25 mg/0.1 ml) were administered in 44 (88%) cases. Pars plana vitrectomy was performed in 16 (32%). The mean baseline visual acuity was 1.395±0.632 LogMAR (Snellen equivalent =6/120), which improved to 0.441±0.553 LogMAR (=6/15) at 60 days follow-up. Cross-tabulation of visual acuity outcome (≤1 LogMAR = good, >1 LogMAR = poor) with demographic and clinical variables indicated an association with lid swelling. No variation in visual outcome was observed with respect to age, gender, type of surgery (phacoemulsification or SICS), or timing of presentation. The addition of intravitreal dexamethasone was not associated with differences in outcome.

## DISCUSSION

This study evaluated visual outcomes and factors influencing the early administration of intravitreal antibiotics in patients presenting with acute endophthalmitis following cataract surgery. The findings corroborate previous research while revealing some differences.

The mean final visual acuity (0.44 LogMAR) showed significant improvement compared to initial postoperative acuity (1.39 LogMAR). This aligns with Rishi et al., who reported a mean final acuity of 0.5 LogMAR following prompt intravitreal antibiotic treatment.^14^ However, our results were slightly better than those of Lalwani et al., who documented a mean final acuity of 0.7 LogMAR, potentially due to differences in presentation timing and disease severity.^15^

In terms of demographic factors, the gender distribution among patients with visual impairment due to endophthalmitis after cataract surgery was nearly identical, aligning with results from a large Korean tertiary care cohort.^16^ Similarly, the variation in age group did not notably affect visual recovery, corroborating Friling et al., who concluded that age alone does not predict post-treatment visual improvement.^17^

Surgical technique, whether phacoemulsification or small-incision cataract surgery (SICS), did not vary much on the visual outcomes in this cohort. While phacoemulsification is generally linked with fewer intraoperative complications and better postoperative vision, direct comparisons in endophthalmitis cases remain limited.^18,19^ Patients who sought treatment within two weeks postoperatively showed better visual outcomes. The Endophthalmitis Vitrectomy Study (EVS, 1995) emphasized the importance of early intervention to optimize prognosis,^20^ but did not specifically analyze timing of antibiotic administration as a continuous variable within the six-week postoperative period. Among clinical indicators, lid swelling, a marker of severe anterior segment inflammation, was found to be more among the poorer visual outcomes supporting Lalwani et al., who identified severe anterior segment inflammation as a negative prognostic factor.^15^

The addition of intravitreal dexamethasone to antibiotics did not significantly improve visual outcomes. This finding coincides with the study conducted by Manning et al. (2018) and Albrecht et al. (2005), who reported no additional benefit from adjunctive steroids in post-cataract endophthalmitis management.^21,22^

Globally, visual prognosis in endophthalmitis varies, influenced by patient demographics and healthcare practices. Visual acuity improvements in this study align with Southeast Asian cohorts prioritizing early intervention and advanced surgical care. However, European studies such as Ohlson et al. report slightly better outcomes, possibly reflecting differences in healthcare infrastructure and patient selection.^23^

This study had several limitations, primarily due to its retrospective design. All cases of presumed infectious endophthalmitis were uniformly treated with intravitreal vancomycin and ceftazidime without standardized selection criteria. Furthermore, potential influencing factors, such as incision type, suturing, and preoperative preparations (e.g., povidone iodine use) were not assessed. The findings may not be generalizable to settings with different surgical practices or antibiotic resistance profiles. The small sample size might lead to limited statistical power to predict the visual outcome at 60 days. Another limitation was the inability to evaluate how organism virulence affected visual outcomes due to unavailable microbiological data. This was attributed to factors like negative culture/stain results, routine antibiotic prophylaxis, and intracameral moxifloxacin use. Although aqueous and vitreous taps were attempted for cultures, they frequently yielded no growth. Pars plana vitrectomy (PPV) remains the most reliable method for obtaining viable samples.

## CONCLUSIONS

In conclusion, lid swelling emerged as a potential predictor of poor visual outcomes, whereas age, gender, type of surgery, timing of presentation, and route of antibiotic administration showed no significant influence. The majority of patients presented within 0-2 weeks after surgery, underscoring the importance of vigilant early postoperative monitoring. Notably, visual acuity improved significantly over time, indicating a beneficial role of intravitreal injections in managing postcataract surgery endophthalmitis. Nonetheless, the small sample size limits the reliability and generalizability of these results.

The findings highlight the value of early detection and timely intervention in optimizing visual outcomes and potentially reducing the need for additional procedures. Future prospective studies with appropriate comparative groups are warranted to strengthen the evidence supporting early intravitreal antibiotic use in this setting.

## Data Availability

The data are available from the corresponding author upon reasonable request.
